# Supplementary material for: Knowledge of Sexually Transmitted Infections and HIV among People Living with HIV: Should We Be Concerned?
Source: Healthcare (Basel). 2024 Feb 6;12(4):417. doi: 10.3390/healthcare12040417 (PMC10888293; doi:10.3390/healthcare12040417)
Supplement: Supplementary file 1 [file healthcare-12-00417-s001.zip › healthcare-2783162-supplementary.pdf]

QUESTIONNAIRE

How old are you? _____ Sex_____________ How do you define your sexual orientation?______________

Region of residency ___________________________

Level of education _____________Employment/job_________________

Year of HIV diagnosis ______

1. What is HIV?
   a) A virus
   b) A bacteria
   c) A fungi
   d) I don't know
2. How is HIV transmitted? (More than one option is correct)
   a) Kiss
   b) Blood
   c) Sexual intercourse
   d) During labour
   e) I don't know
3. Which media is HIV transmittable through? (More than one option is correct)
   a) Saliva
   b) Blood
   c) Vaginal discharge
   d) Sperma
   e) Sweat
   f) Urine
   g) I don't know
4. Is it dangerous to live with a person living with HIV?
   a) No, there is no risk
   b) Sì, if sharing toilet
   c) Sì, if sharing glasses
   d) Sì, if hugging and kissing
   e) I don't know
5. Do you think that a person with HIV who constantly assume their therapy and control the viral load can transmit the infection?
   a) Yes
   b) No
   c) I don't know

---

6. Which is the highest risk age for contracting sexually transmitted infections?
   a) 15-25
   b) 30-40
   c) 40-50
   d) >50
   e) I don't know
7. Does the contraceptive pill protect from STIs?
   a) No, never
   b) Yes, always
   c) It depends on the woman's age
   d) I don't know
8. Which microorganisms are responsible for STIs? (More than one option is correct)
   a) Virus
   b) Bacteria
   c) Protozoa
   d) I don't know
9. How are STIs transmitted? (More than one option is correct)
   a) Saliva
   b) Sperm
   c) Vaginal discharge
   d) Cough and sneezing
   e) I don't know
10. How can HIV and STIs be prevented?
    a) Using condoms during each sexual intercourse
    b) Carefully washing genitals after a sexual intercourse
    c) With regular assumption of contraceptive pill
    d) Practicing coitus interruptus
    e) I don't know
11. In which kind of sexual intercourse is there highest chance of HIV and STIs transmission? (More than one option is correct))
    a) Receptive
    b) Penetrative intercourse
    c) Both
    d) There is no risk
    e) I don't know

---

13. At which age did you have your first sexual intercourse?
____

14. Do you have a regular partner?
    a) Yes
    b) No
    c) I prefer not to answer

15. How many partners did you have in the past six months? _______________
16. If you have had one night stands, how often have you used condoms?
    a) Always
    b) Almost always
    c) Sometimes
    d) Never
    e) I prefer not to answer

---

17. Have you ever received information / education regarding STIs?
    a) Yes
    b) No
18. If yes, where? (More than one option is possible)
    a) Health care staff
    b) Friends
    c) Television
    d) Journals
    e) School
    f) Internet
    g) Family
    h) NGOs
    i) Others:
19. Do you think more educational campaigns regarding HIV and STIs are needed?
    a) No, I think they are useless
    b) No, I think people know enough about it
    c) Yes
20. If yes, which media do you think is the best? (More than one option is possible)
    a) School
    b) Television
    c) Social network
    d) Meet-the-Expert
    e) Paper leaflets
    f) Radio

21. Have you ever heard about U=U (undetectable=untransmittable)?
    a) Yes
    b) No
22. What do you mean by U=U (undetectable=untransmittable)?
a) The person who regularly assume the antiretroviral therapy and have negative viral load (<200 cp/ml) for at least 6 months after first viral negativization, does not transmit the infection to sexual partners
b) The person who regularly assume the antiretroviral therapy and have negative viral load (<50 copie/ml) for at least 6 months after first viral negativization, does not transmit the infection to sexual partner
c) People with HIV transmit the infection, regardless of the viral load, and must always use condoms
d) People with HIV with negative viral load do not transmit any STIs
e) I don't know

23. O you think this survey was a good way to learn new things?
    a) Yes
    b) No

---

24. Have you haver disclosed to your GPs or other doctors (ID specialists excluded)?
    a) Yes
    b) No
    c) I prefer not to answer
25. If not, why? (More than one option is possible)
    a) Fear of judgement
    b) Shame
    c) Sense of guilt
    d) No need
    e) Lack of trust
    f) Other:_____________
26. Have you ever discloed to anyone outside health care facilities?
    a) Yes
    b) No
    c) I prefer not to answer
27. If yes, with who? (More than one option is possible)
    a) Parents
    b) Sons / daughters

c) Siblings
d) Friends
e) Partner
f) Other:___________

28. If not, why? (More than one option is possible)
    g) Fear of been rejected
    h) Fear of judgement
    i) Shame
    j) Sense of guilt
    k) I would not know how to disclose
    l) No need
    m) Other:_____________
29. Sin your opinion, what is needed to fight stigma towards people living with HIV? (More than one option is possible)
    a) Eduaction in schools
    b) Education through social
    c) Education through television and newspaper
    d) Screening campaigns with informative materials
    e) Testimonials by people living with HIV
    f) Other:_____________
30. Did U=U change your self-perception and the opinion you have of the infection?
    a) Yes
    b) No
    c) I prefer not to answer
31. If yes, how?
